# Shoulder biomechanics of para-table tennis: a case study of a standing class para-athlete with severe leg impairment

**DOI:** 10.1186/s13102-022-00536-9

**Published:** 2022-07-24

**Authors:** Pui Wah Kong, Jia Wen Yam

**Affiliations:** Physical Education and Sports Science Academic Group, National Institute of Education, Nanyang Technological University, 1 Nanyang Walk, Singapore, 637616 Singapore

**Keywords:** Kinematics, Variability, Angle, Forehand, Backhand, Statistical parametric mapping

## Abstract

**Background:**

Both able-bodied and Class 7 para-table tennis players compete while standing, but do they use the same techniques to hit the ball? This case study examined the shoulder joint kinematics of a highly skilled para-table tennis player with severe leg impairment.

**Methods:**

One international level Class 7 male para-table tennis player was compared with a control group of 9 male, competitive university team players. Participants performed 15 trials of forehand and 15 trials of backhand topspin drives. Shoulder abduction/adduction angles and joint range of motion (ROM) were measured using an inertial measurement system.

**Results:**

The joint ROM of the para-player was comparable to the control group in the forehand [para-player 38°, controls 32 (15)°] and slightly larger in backhand [para-player 35°, controls 24 (16)°]. Waveform analysis revealed significant differences in the entire forehand drives (p < .001) and the preparation (p < .001) and follow-through phases (p = .014) of the backhand drives.

**Conclusions:**

Coaches should not simply instruct para-table tennis players to replicate the characteristics of able-bodied players. Depending on the nature of the physical impairment, para-players should optimise their movement strategies for successful performance.

## Background

Para-table tennis is a popular event in the Summer Paralympic Games, in which players with physical and intellectual impairments participate. There are 11 player classifications (Class): Classes 1–5 compete in a wheelchair, Classes 6–10 compete standing, and Class 11 is for players with intellectual impairment. Researchers have profiled the eye-hand coordination and executive functions [1] and game characteristics of high-level competitions [2, 3] in different classes of para-table tennis players. Specific to wheelchair para-table tennis, a case study on six players reported that Class 1 players had less functional reach from a seated position than Class 2 players [4]. There are also efforts put in to optimise the design of the wheelchair to improve the players’ functional sweeping area, comfort, and stability [5, 6]. Compared with able-bodied players, wheelchair table tennis players exhibited lower lumbar inversion and pelvic retroversion and slower velocity in trunk rotations due to their limited range of trunk rotational motion [7]. Wheelchair players also employed different upper limb techniques from able-bodied players when executing the forehand and backhand topspin drives [8]. Among well-trained para-table tennis players with intellectual impairment, tactical proficiency related well with spatial visualisation and reaction time [9].

Very little is known about the para-table tennis players in the standing classes (Classes 6–10). Unsurprisingly, standing para-table tennis game characteristics differ from the wheelchair classes [2]. Despite these para-table tennis players competing while standing in the same way as able-bodied players, it is speculated that para-players use alternative biomechanics compared to their able-bodied counterparts to compensate for their physical impairments [10]. Currently, no published studies compare the biomechanics of table tennis between standing players with a disability and able-bodied players. In regular table tennis, previous studies have demonstrated the importance of footwork and the contribution of the upper arm to generate high racket speed [11–14]. Considering that para-table tennis players in standing classes have various degrees of impairments, the kinematics of their movement patterns are expected to differ from those observed in able-bodied players.

The most frequent shots executed in elite table tennis matches are forehand topspin drive (19.5%), forehand topspin counter (16.7%), backhand topspin drive (14.9%) and backhand topspin counter (13.5%) [15]. The forehand topspin drive and counter, characterized by high speed and ball rotation [16], are the most used attacking shots in table tennis. Therefore, players often repeat the forehand and backhand movements hundreds of times to construct stable motion patterns and develop so-called dynamic stereotypes [17]. Ideal motion patterns can help players keep the continuity of hitting, improve overall appearance, maintain efficiency, and conserve energy [18]. Currently, coaches rely mainly on experiential knowledge, informal communication with peers, and theory transferred from able-bodied contexts as primary resources to train para-players. Since most coaches only have prior experiences with able-bodied players, they may not be sufficiently prepared to coach para-players for reasons such as not having appropriate sports science knowledge or adapted physical activity education experience [19]. As technical and tactical skills are crucial determinants for table tennis performance, it is of interest to coaches, performance analysts and medical professionals working with para-table tennis players to understand how elite para-players master such skills.

Considering the small number of para-table tennis athletes with similar degrees of impairment, case study will be a practical approach to investigate selected techniques between players with and without disabilities. Even when performing the same technique (e.g. forehand drives while standing), a para-player may adopt different movement strategies when compared with able-bodied players. The observed differences, if any, may indicate that coaches need to train para- and able-bodied players differently on the same technique, and that injury risk of different body parts can vary across players. In the present case study, the shoulder biomechanics of one Class 7 para-player would be compared to a control group of able-bodied players when executing the forehand and backhand topspin drives. The shoulder joint was chosen because shoulder injuries were of concern in para-table tennis players [20]. In addition, high contact forces at the glenohumeral joint were found during forehand drives in tennis [21], which engaged movement patterns similar to table tennis. Therefore, it was hypothesized that the shoulder joint biomechanics, as reflected by joint angles and range of motion, would differ between the standing para-table tennis player and able-bodied controls when executing forehand and backhand topspin drives.

## Methods

This case study compared one standing para-table tennis player with a group of able-bodied controls by performing two standard table tennis techniques, the forehand and backhand topspin drives. The movement patterns were quantified using motion sensors while waveform analysis and key kinematic variables were extracted for comparison.

### Participants

The present study was approved by the Nanyang Technological University Institutional Review Board (Protocol Number: IRB-2017-08-044). All methods of this study were performed following the Declaration of Helsinki. Written informed consent were obtained from all participants and their legal guardians (for minors) for study participation.

The para-athlete was a 51-year-old male player with 20 years of playing experience. He was a right-handed player adopting the shakehand grip using inverted rubber and long pimples on the front and back surfaces of the racket, respectively. After just 10 month of age, he was diagnosed with poliomyelitis (polio), causing muscle weakness and atrophy, resulting in a smaller left leg. He used a metal knee-ankle-foot orthosis to support his daily living activities and table tennis training and competitions. He is categorized as a Class 7 player due to the severe impairments of his leg. According to the statistics reported by the International Table Tennis Federation for para-table tennis [22], they were 520 Class 7 players worldwide. Among the 26 Class 7 players in Southeast Asian countries, the para-athlete presented in this case study was the only listed player in his country who clinched numerous titles from international competitions. His achievements were bronze in teams Class 7 for 2014 ASEAN Para Games, bronze in doubles Class 10 for 2015 ASEAN Para Games, bronze in teams Classes 6–7 for 2016 Indonesia Para-Table Tennis Open, bronze in singles Class 7 for 2016 U.S. Open, bronze in singles Classes 6–7 for 2017 4th Taichung Table Tennis Open for the Disabled and bronze in teams Class 7 for 2019 PTT China Open. In addition, he also participated in an able-bodied table tennis competition and clinched the first runner-up position in singles for a university staff competition in 2019.

The able-bodied control group comprised nine male competitive university team players [mean (standard deviation); age 23.5 (1.6) years old; playing experience 13.4 (2.6) years]. The inclusion criteria were (1) male, (2) aged between 18 and 35 years old, (3) no impairment, (4) member of a polytechnic or university table tennis team, (5) trained at least two sessions per week in the past three months, and (6) competed in competitions at inter-school or higher level. In addition, participants were excluded if they had any surgery or severe injuries to the upper body in the past six months or were experiencing discomfort or pain at the time of the study. All able-bodied participants were right-handed, adopting the shakehand grip and using inverted rubber for the front surface of the racket. For the back surface, seven used inverted rubbers, while one used short pimples and one long pimples.

### Equipment

**Fig. 1:**
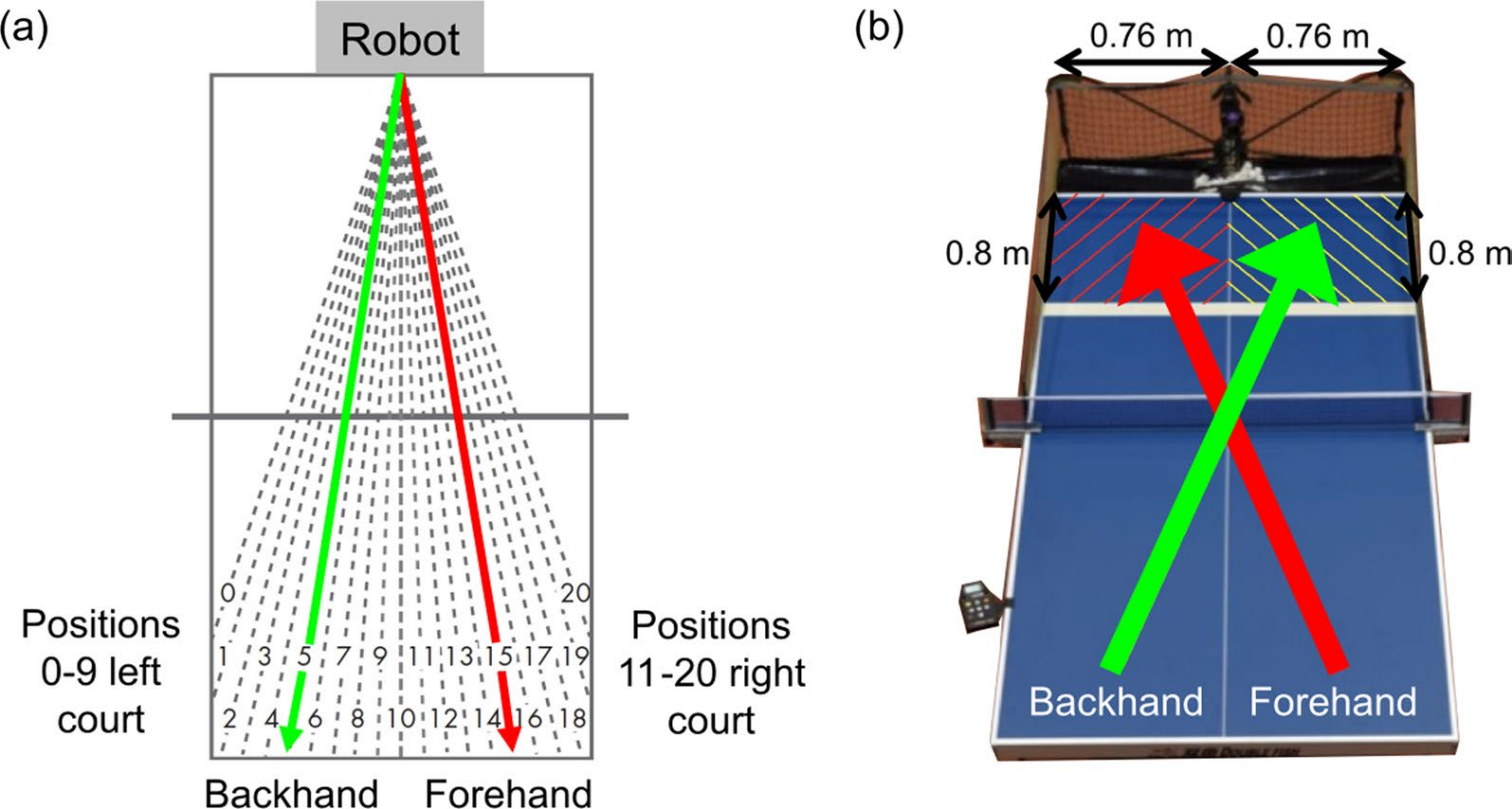
**a** Oscillator positions of Newgy Robo-Pong 2050. Positions 15 (red) and 5 (green) are used for forehand and backhand topspin drives, respectively. **b** Participants were instructed to return the ball diagonally to the marked zone (0.80 m × 0.76 m) in the lower half of the table near the robot

An International Table Tennis Federation (ITTF) Donic Stress table tennis net set (Donic, Germany) and standard D40 + 3 stars table tennis balls (Double Happiness, People’s Republic of China) were used in this study. In addition, the Newgy Robo-Pong 2050 (Newgy Industries, Inc., USA) was used to project table tennis balls to oscillator position 15 for forehand topspin drives and position 5 for backhand topspin drives (Fig. 1a).

**Fig. 2:**
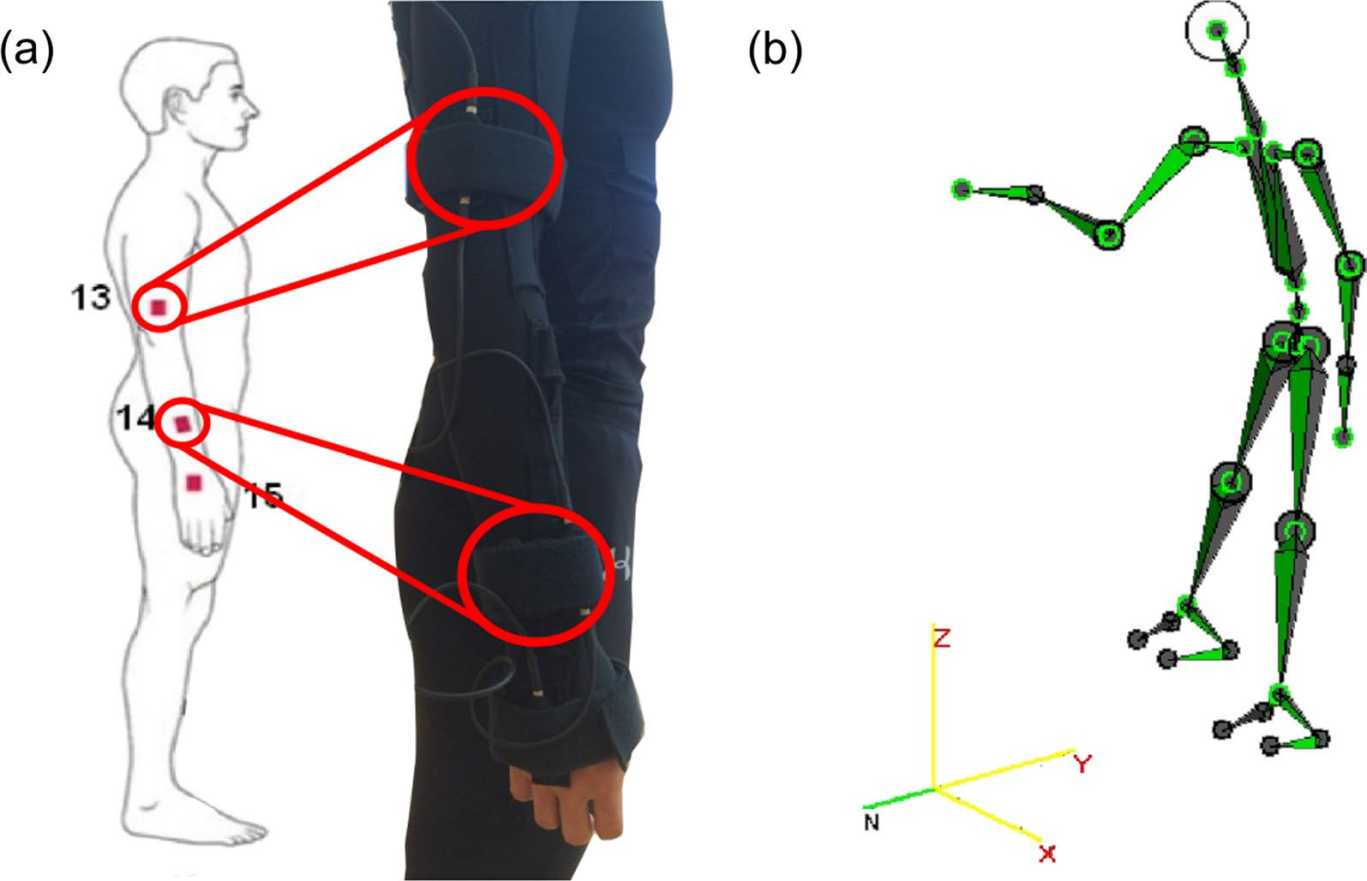
**a** 3DSuit inertial sensors are placed in an adjustable pocket strap aligned to the correct anatomical positions: at the center above the lateral aspect of the elbow joint (sensor 13) and the radius above the wrist joint (sensor 14). **b** Data from the sensors were transferred to a computer and then reconstructed into three-dimensional real-time movements in the AnimaDemo software

The 3DSuit inertial motion capture system (Inertial Labs, Paeonian Springs, VA, USA) captured the shoulder joint motion at a sampling rate of 60 Hz. This system consisted of a Men’s Under Armor HeatGear long sleeve compression shirt and leggings (Under Armour, Inc., USA) mounted with 17 inertial 3D orientation sensors inserted into a fixed pocket over specific anatomical locations (Fig. 2a). For this study, only two sensors (i.e., sensors 13 and 14) of the playing limb were of interest. The inertial sensors are placed in an adjustable pocket strap aligned to the correct anatomical positions: at the center above the lateral aspect of the elbow joint (sensor 13) and the radius above the wrist joint (sensor 14). A Velcro strap was used to secure the sensor positions after a researcher checked that the sensors were placed correctly on the respective anatomical landmarks. According to the manufacturer’s specifications, the 3DSuit has a high accuracy of 1° for angle measurement. When players executed table tennis drives, data from the sensors were transferred to a computer and then reconstructed into three-dimensional real-time movements in the AnimaDemo software (version 11.6, Inertial Labs, Paeonian Springs, VA, USA) (Fig. 2b). The main advantage of the 3DSuit system was that it was a wearable, portable system that allowed data collection to take place outside of the laboratory.

### Procedures

Data collection was conducted at the participants’ usual training venue, and therefore they were familiar with the table used for the tests and the surrounding environment. Participants were asked to don on the 3DSuit before the start of testing. At the beginning of the testing, participants faced south in an initialisation posture to calibrate the 3DSuit system. This posture required the participant to stand in the anatomical position with face directed forward, arms at the side with palms facing forward, and feet shoulder-width apart. After successful calibration, participants were given up to 5 min to warm up and practice driving balls projected by the robot. Then, they were allowed to hit 30 consecutive forehand and 30 consecutive backhand topspin drives.

The tests commenced after the familiarisation period. The order of executing forehand or backhand first was randomly assigned. Participants performed 30 trials (3 sets of 10 consecutive drives) for forehand topspin drives and 30 trials (3 sets of 10 consecutive drives) for backhand topspin drives. Sufficient rest time was allowed between each set and between the two types of drives to prevent fatigue. They were instructed to return the ball diagonally to land on the lower half of the table within the marked 0.80 m × 0.76 m zone (Fig. 1b). Returns were deemed valid when the ball landed diagonally within the target zone [8].

The time histories of shoulder joint abduction/adduction angle for all table tennis strokes were determined from the 3DSuit system. Each stroke can be split into (1) preparation phase and (2) execution and follow-through phase [16]. The athlete swung the racket backward during the preparation phase to get ready for the drive. During the execution and follow-through phase, the player drove the racket forward to hit the ball and then slowed down. The beginning and end of each phase were visually identified by the same researcher (JWY) based on the wrist position.

### Data processing

Shoulder joint angle data were filtered at 8 Hz using a low-pass 4th order zero-lag Butterworth filter in MATLAB (R2017b, MathWorks, USA). This cut-off frequency was chosen based on a previous study examining the shoulder kinematics of tennis forehand topspin drives [21]. Although the participants executed 30 trials for each drive, some were invalid because the ball landed outside the target zone or did not land on the table. Since all participants had executed at least 15 valid trials in both forehand and backhand topspin drives, we standardised to analyse the first 15 valid trials per subject for good consistency. The joint angle data were time normalised (0–100%) for each trial. An average shoulder angle-time history of the 15 trials was derived to represent the general technique of each participant. This practice of taking an average time-history of multiple trials was in line with the current practice in biomechanical analysis of sports movements, including those on table tennis [8, 23, 24]. Shoulder joint range of motion (ROM) was calculated as the excursion from the instant of maximum shoulder abduction to the end of the drive.

### Statistical analysis

Descriptive statistics of the shoulder joint ROM in the able-bodied control group were calculated as mean (SD). Then, the para-player’s joint angle-time histories were compared to the controls using one-dimensional statistical parametric mapping (SPM, one-sample t-test). SPM allowed analysis of the entire waveform over time, offering a more comprehensive analysis over extracting discrete kinematic variables at critical events such as maximum or minimum values [25]. Such waveform analysis can provide additional dynamic information that may be missed when reporting discrete variables (e.g., joint angles, ROM) alone [26]. Specific to table tennis, SPM1D analysis revealed differences between men and women in the contribution of thoracic rotation, external shoulder rotation, dorsal flexion, and supination in the wrist during the hitting phase of topspin backhand strokes [23]. More recently, SPM1D analysis identified different kinematics between Polish and Chinese female table tennis players when executing the topspin backhand strokes [24]. The authors attributed the differences to the two countries’ training methods and coaching systems. In this present study, the SPM1D analysis was performed to compare the para-athlete to the control group in MATLAB (R2017b, MathWorks, USA) using the codes downloaded from www.spm1d.org. The significance level for statistical tests was set at 0.05.

## Results

**Fig. 3:**
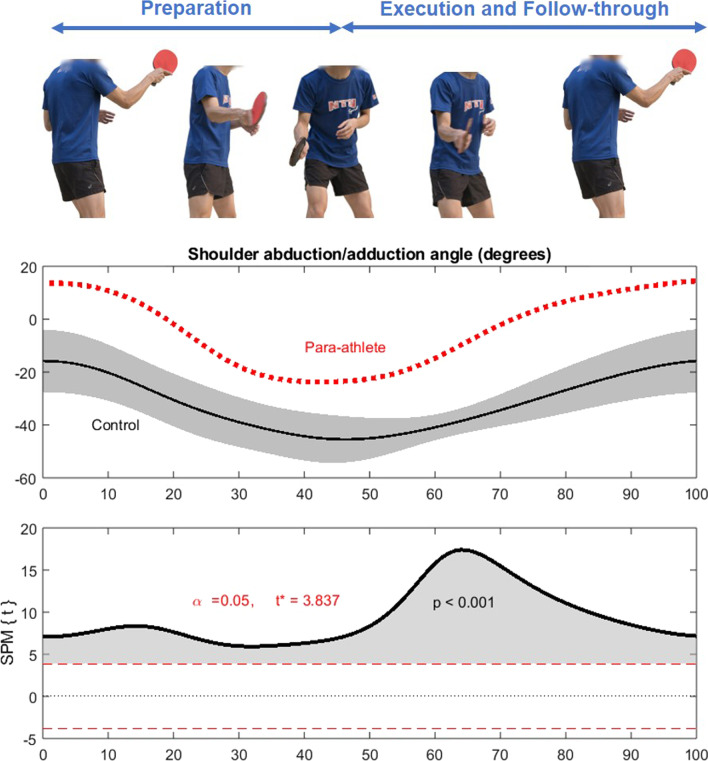
Shoulder abduction/adduction angles during table tennis forehand topspin drives between the Class 7 para-player (standing) and able-bodied controls (n = 9). A positive and increasing angle indicates shoulder adduction, while a negative and decreasing angle indicates shoulder abduction. *Note*: The person shown in this figure is co-author JWY who acted as a model to illustrate the movement

**Fig. 4:**
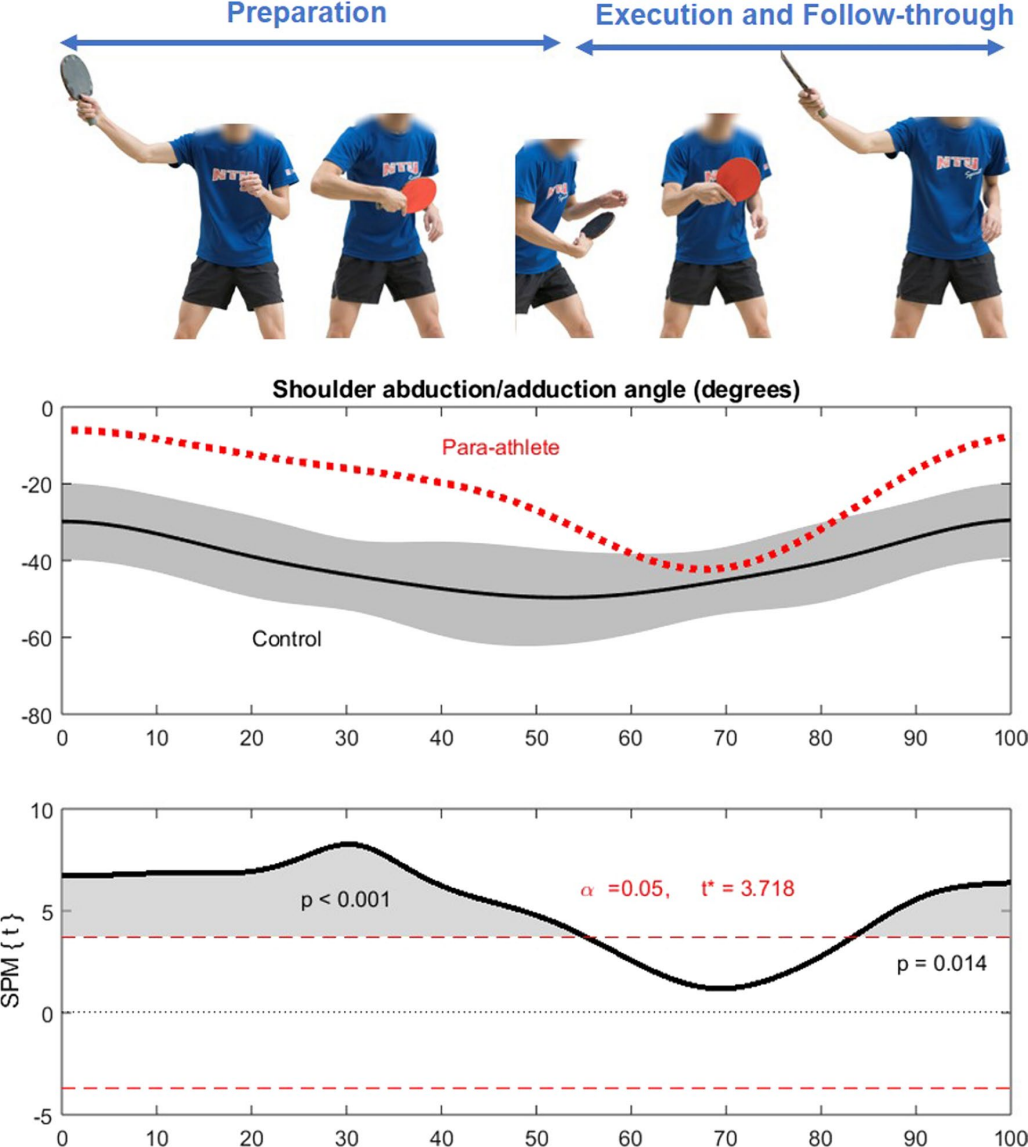
Shoulder abduction/adduction angles during table tennis backhand topspin drives between the Class 7 para-player (standing) and able-bodied controls (n = 9). A positive and increasing angle indicates shoulder adduction, while a negative and decreasing angle indicates shoulder abduction. *Note*: The person shown in this figure is co-author JWY who acted as a model to illustrate the movement

For forehand topspin drives, the shoulder abduction/adduction ROM was 38° in the para-player and 32(15)° in the control group. SPM analysis revealed significant differences in the entire waveform (p < 0.001) from the beginning to the end of the drive (Fig. 3). For backhand topspin drives, the shoulder joint ROM was 35° in the para-player and 24(16)° in the control group. The para-player exhibited significantly different movement pattern in the preparation (p < 0.001) and follow-through phases (p = 0.014) of the drive (Fig. 4). The person shown in Figs. 3 and 4 is co-author JWY who acted as a model to illustrate the movement.

## Discussion

This case study compared the shoulder joint biomechanics between a Class 7 para-table tennis player and able-bodied controls when executing table tennis movements. For forehand topspin drives, the shoulder abduction/adduction angles distinctly differed between the para-player and controls throughout the entire movement. Backhand topspin drives also differed substantially, with the para-player displaying greater shoulder abduction in the preparation and follow-through phases than the able-bodied controls.

The present study is the first to report the shoulder biomechanics of para-table tennis competing while standing to the best of the authors’ knowledge. The findings provide empirical evidence to confirm the previous speculation that para-players would use alternative biomechanics to compensate for their physical impairments [10]. In the present study, the Class 7 para-player executed both forehand and backhand topspin drives using distinctly different movement patterns than the university team players (Figs. 3 and 4). The same phenomenon was observed in other sports comparing between para and able-bodied athletes. Bjerkefors and colleagues [27] investigated 41 para-kayakers, and 10 able-bodied kayakers competing at the international level found that both groups had different kayak paddling techniques. Para-kayakers from Kayak Level 1, 2, and 3 demonstrated significantly larger upper body joint angles (e.g., greater maximum shoulder extension, flexion ROM, maximum abduction, and rotation ROM). I﻿n comparison, able-bodied kayakers demonstrated significantly larger lower body joint angles (e.g., greater trunk and pelvis rotation angles, hip and knee flexion ROM, ankle dorsiflexion, and ankle flexion ROM). In this case study alone, it is impossible to confirm the extent of the anatomical impairment on movement techniques since there can be a wide intra-individual variance among individuals diagnosed with polio. As the player in the present study is a high-performance athlete with numerous achievements, he may have adapted an effective movement strategy that works well for his anatomical and functional limitations. This speculation, however, should be confirmed by future research considering more factors such as strength, joint range of motion, functional reach, reflex measure, and balance ability of the para-athletes.

An important implication arises from the present study: para-table tennis players should not regard the movement patterns of able-bodied players as the reference standard and simply copy their movements. Depending on the nature of the physical impairment, para-athletes should optimise their own movement strategies for successful performance. According to Taylor and colleagues’ 400 m freestyle swimming study [28], the race pacing strategy adopted depends on the swimmer’s physiological, biomechanical, and psychological considerations. For example, the parabolic fast start strategy was adopted only by para-swimmers, while the fast start strategy was adopted only by able-bodied swimmers. The assumption that the para-table tennis players can effectively adapt able-bodied table tennis skills and techniques may not be appropriate. Therefore, coaches should not train or instruct para-table tennis players’ techniques to replicate the characteristics of able-bodied table tennis players. Although many movement strategies exist, some are more efficient and effective than others. Therefore, the optimal technique for para and abled-bodied table tennis players may differ. Future research is warranted to search for desirable techniques for para-table tennis players with various types and degrees of impairment.

Using a case study approach, the present study demonstrated that both para- and able-bodied players could achieve successful forehand and backhand topspin drives execution using rather different movement patterns. Therefore, it will be logical to use a different pedagogical approach to train players with and without physical disabilities. For example, Dehghansai and colleagues [29] introduced Newell’s constraint-led model and its multidimensional spectrum and practical scope to address the complexities of athlete development in para-sport. To provide specific context to coaches, additional sub-categories under each of the three overarching constraint categories (i.e., individual, task, and environment) may be necessary to capture nuances associated with para-sport. Guided by this constraints-led approach, Pinder and colleagues [30] simplify task designs to improve the long jump technique for a para-long jumper. As a result, they managed to improve the technical execution of the drive and leg extension and increase the alignment of the athlete's perception with movement. Extending from the present case study, future research can explore the application of constraint-led model to coach para-table tennis players with consideration of each player’s individual needs.

In other studies on able-bodied table tennis, Xia and colleagues [31] reported similar shoulder abduction/adduction angles between players performing forehand topspin drives using shakehand and penhold grips. Others emphasized the importance of proximal-to-distal sequencing of the playing limb to generate high racket speed in forehand drives [23, 32]. However, it should be noted that most previous studies only compared selected kinematic variables at critical events such as initial position, contact, and follow-through phases [11–14, 23, 31, 32]. The present study examined the entire waveform on the drive, including preparation, contacting, and follow-through phases using SPM analysis. This new approach provides a more comprehensive understanding of the player’s technique as a continuous movement rather than analysing a few key events. In addition, the use of an inertial measurement system allows data collection to be conducted at the players’ usual training venue, which is more ecologically valid than testing in the laboratory.

There are limitations to the current study. As a starting point, this case study examined only the shoulder joint kinematics based on previous concerns in shoulder injuries among para-table tennis players. Future studies can expand the biomechanical measurements to include other body parts such as the elbow, wrist, torso, and lower limbs to better understand the playing techniques of para-players. The sample size is another major limitation since there are very few para-players in our country. We acknowledged that the Class 7 para-player in the present study might not represent players in the same or other classes. Given that each para-player likely has a unique medical history associated with the impairment, a single case study remains insufficient to make robust conclusions. Future work should remain open to individualised approaches and to obtain generalisable knowledge for different sub-groups of para-players.

## Conclusions

This case study showed that the Class 7 para-player displayed different shoulder joint kinematics when performing table tennis forehand and backhand topspin drives than the able-bodied players. These findings suggest that para-players and coaches should not simply replicate the movement patterns of able-bodied table tennis players. Instead, depending on the nature of the physical impairment, para-players should optimise their own movement strategies for successful performance.

## Data Availability

The datasets used and analysed during the current study are available at the NIE Data Repository: https://doi.org/10.25340/R4/TDIAJS.
